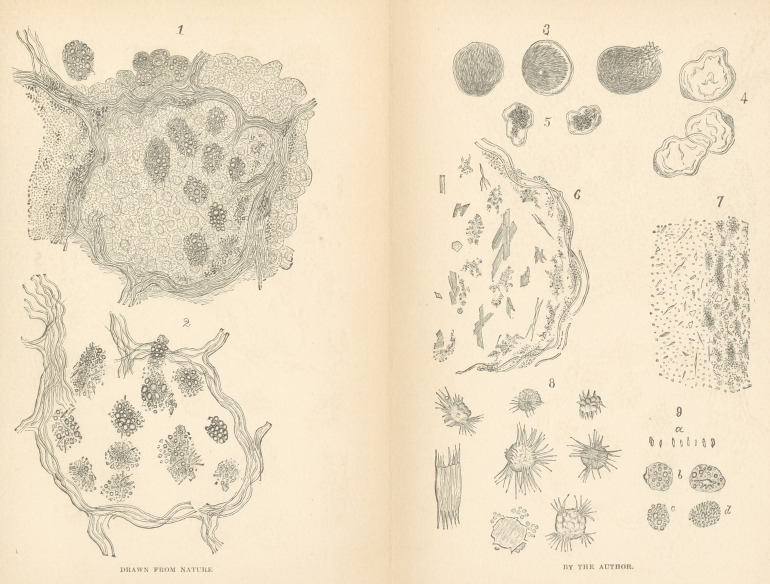# Microscopical Investigation into the Nature of the So-called Bacillus Tuberculosis

**Published:** 1882-12

**Authors:** H. D. Schmidt

**Affiliations:** Pathologist to the Charity Hospital of New Orleans; New Orleans


CHICAGO MEDICAL

JOURNAL & EXAMINER.

VOL. XLV.—DECEMBER, 1882.—No. 6.

### Original Communications.

ARTICLE I.

### MICROSCOPICAL INVESTIGATION INTO THE NATURE OF THE SO-CALLED BACILLUS TUBERCULOSIS.

By H. D. SCHMIDT, M.D., Pathologist to the Charity Hospital of New Orleans. Read before the Orleans Parish Medical Society, Nov. 6, 1882.

Koch's discovery of the bacillus tuberculosis, published in the "*Berliner Klinische Wochenschrift*," April, 10, 1882, renewed the interest which I had previously felt in this subject, whilst engaged, during the fall and winter of 1881, in extensive microscopical investigations into the structure, the development, and degeneration of miliary tubercle. Though my faith in the theory of the *contagium vivum* had been considerably shaken by my former failure to detect minute parasitic organisms in the blood and tissues of fatal cases of yellow fever and leprosy, nevertheless, this theory once more gained my confidence by the authoritative position of the discoverer of this new bacillus. But a certain point in his own statements appeared to me rather suspicious. I refer to the statement that the bacillus tuberculosis differed from all other known bacilli, with the sole exception of the bacillus lepræ, in its behavior toward staining fluids. Still, not regarding this special behavior as impossible, I determined to regard the existence of this bacillus as a fact, unless convinced of the contrary by personal observations.

In order, therefore, to inquire into the existence and nature of this new bacillus, I went, at once, to work, and prepared, from the abundant pathological material constantly at my disposal, a number of specimens of sputa, and thin sections of tuberculous lungs, which sections I stained with the methyl-violet and vesuvin liquids, prepared with the greatest precision after Koch's directions, and then mounted them in Canada balsam. In examining both sputa and sections under the microscope, I failed, however, to detect anything in the shape of a bacillus, stained blue *to the degree which I expected.* Thinking that, perhaps, the brown color of the vesuvin might have hidden the bacilli, I now concluded to lay aside this color, and, accordingly, stained another set of sections and specimens of sputa, with methyl-violet alone, the surplus of which I removed by absolute alcohol and oil of cloves. Some of these preparations were then mounted in balsam, and the rest in glycerine. The microscopical examination, however, was attended by the same results as before, with the exception of one specimen of sputa, in which I observed a few minute, highly refractive rods of a greenish-blue tint, resembling in shape small bacilli.

Although I had thus far failed to demonstrate satisfactorily the bacillus tuberculosis, I still had faith in its existence, because I had learned from some medical journals that the minute, blue-stained rods had really been seen in London in some preparations which Koch himself had sent to that city; while, on the other hand, the want of success in staining the organism, experienced by some English microscopists at the Brownian Institute of the same city, consoled me for my own failure. But being still very anxious to behold this new parasite, especially in its original haunts, miliary tubercle, I resolved to have recourse to the old, long-tried bacteria detective, a solution of caustic potassa. With this solution, 30 per cent. in strength, I then succeeded in rendering visible, in thin sections of the tuberculous tissue, as well as in specimens of sputa, a number of minute rods, corresponding in form and size to those which Koch described as the bacilli tuberculosis. They were, as this investigator had stated, generally met with in small groups along the margins of the tubercles, exhibiting a greenish-blue tint, and being highly refractive. Notwithstanding that this high degree of refraction, exhibited by the bacilli, appeared to me extraordinary, I could not deny that, in other respects, the rods which I observed presented somewhat the form of these minute organisms, and this view became strengthened when I observed a minute granule on one end of one of the rods, which I imagined to represent the sporangium, also reported to have been discovered by Koch. And, having thus arrived at the point of admitting to myself that these bodies might represent true bacilli, I was ready to set aside my former views on this subject, and to pass over into the camp of the germ-theorists. This change of opinion, however, lasted but a short time, for a certain observation which I then made in the same direction, determined me to remain for awhile in the old quarters. I observed that these rods were almost always found in company with fat globules, and frequently, also, with granules of black pigment. This observation, of course, changed the face of the question, for it reminded me of the fatty crystals which I had observed, not only in the degenerating fat-cells of the subcutaneous layer of the skin in cases of leprosy, but also in the cells of other pathological tissues, as well as in vomited matter, and in fæces. In fact, every working pathologist knows that the crystals of fatty acids, either free or enclosed in cells, are not infrequently encountered in the examination of pathological specimens.

In returning, therefore, to the section of tuberculous lung-tissue under discussion, it remains to be stated, that at the time when I first beheld the groups of minute rods, the protoplasm of the tubercle-cells in the section had already been considerably acted upon by the solution of potassa, and thus been rendered very clear. In examining the next section, likewise treated with the solution of potassa, I endeavored to find the rods, before this fluid should have rendered the histological elements of the lung-tissue indistinct; and while thus examining along the margins of the tubercles in the section, I noticed a number of cells of an opaque and somewhat blackish appearance, and in keeping my attention directed to these cells, I observed how their protoplasm cleared up more and more, and, finally, that they were filled with larger and smaller fat-globules, between which the minute rods gradually made their appearance (fig. 1). With the clearing up of the protoplasm, the latter were rendered sufficiently distinct to bear a close examination. In this section the cells, which were almost all filled with fat-globules, and many of them with pigment-granules beside, chanced to contain numerous rods, which, however, considerably differed among themselves, both in length and form. While some of them represented true rods, with slightly pointed, or even square ends, others presented the form of fine needles, with their ends terminating in fine and very slender points; and others, again, presented the form of a lancet. All these minute bodies, however, were characterized by very sharp and dark contours, and by refracting the light very highly. L ke fat-globules, they concentrated the light passing through them when brought out of focus by removing the objective further off, while they were rendered dark by a closer approach of the latter.

These examinations were then repeated on a great number of other sections, which I had specially made for these investigations from the lungs of four cases of tuberculosis, including two of miliary tubercle. But, in every section which I examined, I met with the same results as above stated. In a number of specimens of sputa, also treated with the solution of potassa, the minute rods were now easily detected, either free, or still enclosed in the fattily degenerated, and frequently pigmented cells.

The suspicion, however, that these bodies might, after all, only represent minute fat-crystals, had taken hold of my mind, and was much increased by the following accidental observation: From my microscopical examinations of the various organs of several patients dead of yellow fever which had occurred during the summer at the Charity Hospital, I had kept a large number of thin sections for subsequent studies, more especially for the detection of minute parasitic organisms. And it thus happened that, at the same time when I made the above described observations on the sections of tuberculous lung-tissue, I also treated with the same solution of caustic potassa, for the purpose of detecting bacteria, some sections of yellow fever liver, kidney, and heart, which, as I knew from previous examinations, were considerably affected with fatty infiltration or degeneration. But failing to discover any bacteria in these sections, I put them aside, mounted as they were. The next morning, however, when I chanced to re-examine them under the microscope, I was surprised to find them covered with crystals of fatty acids, which had formed since the preceding day. The crystals themselves, as well as their arrangement into figures, appeared to differ in the different tissues represented in these sections; for, while in one section they appeared in the form of a regular cluster of needles, radiating from a common center, they presented themselves in the other in the form of branches springing from a delicate stem, or even in small groups, arranged in an irregular manner. The greater number of these crystals resembled those of margaric acid; they were sword-shaped, and were marked by very delicate outlines; others appeared in the form of minute needles, or lancet-shaped leaflets, with very dark and heavy contours, forming ramifications like the branches of a tree; these, very likely, represented stearic or some other fatty acid. At any rate, some of the individual minute crystals, especially those at the root, where the little branch arose from the patch of fat, were identical in form with the minute rods, needles and leaflets contained in the tubercle cells. In the sputa of tuberculous patients, treated with the same potash solution, the same crystals above described were also observed to form (fig. 8). Subsequent investigations into this subject showed me, furthermore, that these crystals may be produced in any tissue which has undergone fatty degeneration, such as cancer, etc.; and even with an alkaline solution much weaker than the one which I had used. If a film of tallow, produced by rubbing the glass slide gently with a small piece of tallow candle, is treated with caustic potassa, minute lancet-shaped crystals will appear in a time as short as half an hour.

The artificial production of pseudo-bacilli tuberculosis in the form of minute fat-crystals, which I had learned by this observation, could not but strengthen the suspicion with which I had previously regarded the minute rods and leaflets contained in the tubercle cells, and therefore, all that now remained to be done was to prove, in a more reliable and positive manner, that these bodies also represented the crystals of a fatty acid. Accordingly, I selected several sections, cut from the tuberculous portion of a lung which I knew, from previous examination, to contain considerable numbers of the degenerated cells in question. These sections, in order to be sure of their really containing these cells with rods, were treated with the potassa solution, and then put under very thin cover-glasses; upon glass slides, for examination. Having thus assured myself of the presence of numerous rods and leaflets in the cells of these sections, I placed the slides, upon which the latter were mounted, under water contained in glass saucers, for the purpose of making the cover-glasses float away from the sections. As the latter, by the action of the alkaline solution, were rendered quite soft, the manipulation had to be executed with great care. Nevertheless, I fully succeeded in removing the delicate sections from the slides into the water. From this, they were, by the aid of a small camel's hair brush, carefully transferred to alcohol, and from this to ether, contained in a small test-tube. After the ether had been heated to its boiling point, for the purpose of dissolving, if possible, the minute rods in the sections, the latter were transferred again to the alcohol, and from this to the water, to be finally remounted upon the slides in a weak solution of potassa, or in a medium representing a mixture of alcohol, water and glycerine. Upon close re-examination of these sections, under the microscope, it was found *that every minute rod and lancet-shaped leaflet which they had before contained, had now disappeared;* in other words, *they had been dissolved by the ether.* The results of this experiment speak for themselves, for they conclusively show that my suspicions respecting these minute rods and leaflets, *that they represented the crystals of a fatty acid*, were well founded. True bacilli, as everybody knows, will never dissolve in ether; on the contrary, their protoplasm will only be rendered more consistent by the action of alcohol and ether. I obtained the same results with other sections, which I subjected to the same process, with the modification that instead of boiling the ether, I left them in cold ether for several hours.

Having thus become familiar with the characteristic appearance of these minute crystalline rods and leaflets, and, moreover, knowing now the particular localities in the sections where to find them, I proceeded to re-examine those sections which, stained by Koch's method, I had mounted in Canada balsam. These re-examinations ended in the discovery of a number of minute, faintly blue rods, identical with those observed in the sections treated with the solution of potassa. In these sections, however, the pseudo-bacilli appeared less numerous, for the reason that the majority of them remained hidden in the protoplasm of the cells, rendered brown by the vesuvin, and therefore, only those rods which happened to be outside of the protoplasm, or in very thin and light portions of the section, could be seen; the great transparency of the Canada balsam, also, prevented them from being brought into view. But the principal reason for the failures in detecting the imaginary bacilli during my first examination of these stained sections was, that I had expected to find them stained, *like true bacilli, or micrococci, of a bright blue*, while, in reality, *they are not stained at all*, but simply present their natural bluish tint.

While I was thus engaged in these investigations, I learned, through some medical journals, of the improved staining methods of *Ehrlich* and *Gibbes*, and took steps to procure from New York some aniline oil and chrysoidin, which could not be obtained in this city. As soon, therefore, as these substances came to my hands, I prepared, in accordance with the given directions, and with the greatest precision, the liquids required for these methods, and proceeded to put them to test. And, in strict accordance with the prescriptions for these two new methods of staining, a number of sections of tuberculous tissue, and specimens of tuberculous sputa, were stained, and then mounted in Canada balsam. But, as was the case with the preparations stained by Koch's method, the examinations of these specimens led to the same results. The pseudo-bacilli remained unstained, presenting only, as before, their natural bluish tint.

After this new failure experienced in the attempts at staining the imaginary bacillus tuberculosis by the improved methods of Ehrlich and Gibbes, which, on account of the aniline oil contained in the staining fluids, were represented as much superior to Koch's original method, I concluded to inquire a little closer into the nature of these fluids, and accordingly, examined under the microscope a drop of Ehrlich's solution. The examination revealed the fact that this fluid contained an indefinite number of minute globules of the aniline oil, colored blue by the methyl-violet; a circumstance which at once exposed the erroneous idea of the specific action of this oil in the staining of these bacilli. This substance, representing the basis of the aniline colors, was supposed to prepare the bacilli for the reception of these colors. The results which I obtained from the microscopical examination show that, though a certain quantity of the oil may be absorbed by the water, another, and perhaps a larger portion remains—notwithstanding the close filtering—suspended in the fluid in the form of minute globules, which, in reality, are the true carriers of the color. Now, it is hardly possible that these colored oil-globules are absorbed by the minute crystalline rods, leaflets and needles. The examination of Gibbes' staining fluid, in which the aniline oil is dissolved by alcohol instead of water, gave the same results.

But again, in both of these methods, the specimens, after being stained, are treated by a solution of nitric acid, until, according to the directions given, the color absorbed has nearly disappeared, when the action of the acid is arrested by washing in water. In order to see how much was left of the color in the sections, I selected a number of them, treated to a greater or lesser extent by the acid solution, and mounted them in different media, such as water, glycerine and Canada balsam, for microscopical examination. The latter showed that the color which had remained behind was not fixed in the pseudo-bacilli, but mainly in the nuclei, and also protoplasm of some of the cells. The results of this examination, therefore, showed that the advantage gained by treating the specimens with the solution of nitric acid is, like that of the aniline oil, but imaginary; and furthermore, that the specimens, after being stained, might as well be treated at once with the vesuvin, or chrysoidin, as Koch originally did. In fact, the method of staining which Koch originally employed is, if not better, at least fully as good, as those of Ehrlich and Gibbes.

In the staining of the bacillus lepræ, stress has been laid upon treating the leprous tissue, previous to the staining, with an alkaline solution, particularly of caustic potassa. The pretended purpose of this alkaline treatment is to dissolve the outer layer of these bacilli, supposed to consist of cellulose, and to facilitate, by the destruction of this layer, the absorption of the aniline colors by these organisms. This supposition must appear unfounded to any one familiar with the staining of vegetable tissues, as vegetable cells, the most of which possess an outer cellulose coat, absorb very readily aniline colors, especially methyl-violet, without being subjected to the action of an alkali. Koch also attaches the greatest importance to the presence of an alkali in the staining fluid for his bacillus tuberculosis, but omits to state the object in view. He, however, says distinctly, "that by increasing the strength of the potassa solution, the bacteria may still be stained in places, where with a weaker solution of this alkali they refuse to make their appearance."* Now, by referring to the description which I have given above of the process by which the minute rods, or pseudo-bacilli, are rendered visible, every thinking physician will at once guess the part which the solution of caustic potassa plays in the demonstration of these bodies. This part simply consists in rendering the protoplasm of those degenerated cells, containing the rods, softer, and clearer to the passage of light, by which the rods are finally brought into view. In the methods of Ehrlich and Gibbes, the same part is played by the aniline oil.

Besides the stained sections already mentioned, I have, in the course of these investigations, stained many others, either by the methods of Koch, Ehrlich and Gibbes, or by modifying them to a certain extent; and I have thus ascertained, as I previously expected, that the successful staining of the pretended bacilli does not depend upon a given strength of the staining fluid. Every body knows that in the staining of animal and vegetable tissues, a weak solution of the staining material stains as thoroughly, or even more so, than a stronger one, provided the tissues are left in it a sufficient length of time. The weaker the solution, therefore, the more time is required by the tissues to absorb the color, and *vice versa*. The staining of Koch's bacillus tuberculosis

* "*Berliner Klinische Wochenschrift*," April 10, 1882, p. 222.

takes place under the same conditions. Neither is it essential, as in Koch's method of staining, that the alkali should be incorporated in the staining fluid; on the contrary, if the sections are first exposed to the action of a one per cent. solution of caustic potassa — which is about equivalent to the alkalinity of Koch's staining fluid—for twenty-four hours, and subsequently to the simple solution of methyl-violet, they will absorb the color just as well, and more rapidly, with the same final results. As long as the alkali fulfills its mission of softening the protoplasm of the cells for the ready absorption of the coloring material, it matters not whether the staining be accomplished in a shorter or longer time; though I think that the tissues are stained more evenly and thoroughly by leaving them for a longer time in a weaker solution of the staining material.

In connection with the foregoing, I will mention an observation, which I made on some sections of tuberculous lung, soaked in pure aniline oil, before they were put in the solution of methyl-violet, and which, after being stained by the latter, were treated with the solution of nitric acid until rendered green, and then mounted in glycerine. The microscopical examination of these sections showed nothing particular. But, when re examining them on the next day, I found them covered with a great number of larger and smaller brown fatty globules, from the periphery of which projected a number—from one to four or five, of long needle-shaped crystals; the thickness of the needles was proportionate to the size of the globules, some of which were very minute. Beside these needles arising from the fat-globules, however, there were a number of others, more minute and slender, and without globules, but lying free in the field, either single or in small groups; they differed in length, some terminating in very fine and slender points, while others appeared in the form of minute rods. I of course perceived at once that these globules in the section represented aniline oil in the act of crystallization, which was probably induced by the conjoined influence of the nitric acid and the tissues. Next, I put a drop of Ehrlich's solution upon a glass slide, and after somewhat flattening it, put into its center a minute portion of the nitric acid solution, which at once rendered this part of the staining fluid green; then a cover-glass was applied to the preparation, and the slide put aside. When the preparation was examined on the next day, minute needle or rod-shaped crystals were observed. These facts show that minute crystals of various forms may be artificially produced in animal tissues with great facility.

At this period of my investigations, as may be judged from the above statements, I was convinced of the crystalline nature of the minute rods which I had observed in the sections of tuberculous lung; and accordingly, I might have published the results which I had obtained about three months ago. But, even at the risk of losing my claims to the priority of these observations, I preferred to extend the investigations to a still greater number of cases, in order to substantiate the facts which I had elicited. Therefore, the investigations were repeated on eight additional cases of tuberculosis of the lungs, of which I made hundreds of sections, which, together with numerous specimens of sputa, were carefully prepared and studied in the various manners already described. Thus the experience I gained on the subject is now based upon the careful examination of several hundreds of sections from tuberculous lungs, and specimens of tuberculous sputa. Beside these preparations, however, I examined a great number of sections already stained with picro-carmine and hematoxylin, which I had kept in good condition in alcohol and water from my previous studies on the structure of the miliary tubercle during last winter. These sections proved, notwithstanding their being stained, to be as useful for the study of the subject as those freshly made.

Having stated, in the preceding pages, the principal facts elicited by my investigations into the nature of the so-called bacilli tuberculosis, I shall now proceed to describe and discuss in detail some of their more salient points, commencing with the minute crystalline rods, leaflets and needles, somewhat resembling in form, when superficially examined, the bacilli.

As I have mentioned once before, these bodies (figs. 1, 2 and 3) differ among themselves, both in form and size, and are distinguished by refracting the light in a much higher degree than true bacilli. When, therefore, a very thin section containing a number of these rods is examined under the microscope, with the aid of a first-class achromatic condenser, it will be found that those rods which lie at the surface of the section appear, *like other crystalline bodies*, very bright and shining, as soon as they are illuminated by very oblique rays of light proceeding from two different directions—obtained by making use of the eccentric orifices in the diaphram of the condenser—while, at the same time, all the other elements of the section appear but feebly illuminated. Although the different forms of these bodies may already be recognized without difficulty in the sections cleared up by the solution of potassa, they are still better seen by allowing the potassa to act for a longer time upon the histological elements of the section, until these are nearly destroyed with the exception of the elastic tissue framework of the alveoli. Then, by passing a small current of water between the slide and the cover-glass by means of a small piece of blotting-paper, the disintegrated protoplasm is washed away, and the minute crystals are properly exposed for examination (fig. 2). In such preparations, the fine, slender points of the needle or sword-shaped crystals, which before were buried in the protoplasm, are now fully exposed to view. A number of these pseudo-bacilli will present themselves in the form of shorter or longer ellipsoid or lancet-shaped bodies, while others will appear in the form of longer or shorter rods. The ends of many of the latter are distinctly rectangular, while in others they are slightly pointed. There are, however, a number of these rod-like crystals, particularly the shorter ones, which only exhibit their lateral outlines, in the form of two dark parallel contours, while the outlines of their rectangular ends can hardly be seen (figs. 1, 2 and 7). In these rods, the ends probably terminate in sharp edges, or are buried in the remains of the disintegrated mass of protoplasm.

The crystalline pseudo-bacilli are almost always found in company with a larger or smaller number of fat-globules, together with black pigment-granules, either single, or collected into small masses. Sometimes small groups of crystals are met with only associated with pigment, or, if fat be present, it is in the form of a few minute globules; but not unfrequently, also, single or small groups of crystals are found only accompanied by a small group of pigment granules (figs. 1 and 2). In fact, the association of these minute crystals with fat or pigment is so characteristic, that in order to find the former in the sections, it is only necessary to look for the groups formed by the latter; for it is very rare to find even a single one of these minute crystals without some pigment-granules in its vicinity. In sections mounted in oil of cloves or Canada balsam, the fat disappears from view, in consequence of the transparency of these media, while the pigment-granules, on account of their dark contours, are rendered more distinct. The centers of these granules, when brought into the proper focus, present the same bluish tint as characterizes the pseudo-bacilli. And, as far as I may judge from my observations, there remains no doubt that the fat-globules, crystals, and pigment-granules stand in a very close relation to each other. This supposition is not only supported by the fact that these elements are almost always found associated together in groups, but moreover, by other forms observed, which are transitory between these fat-globules, crystals, and pigment-granules. Thus, in closely examining many of the collections of black pigment deposited along the margins of the miliary tubercles, or even along the blood-vessels in the alveolar septa, small collections of fat, not in the regular form of globules, but in that of triangular, quadrilateral, elongated, or otherwise irregular patches of a dirty greenish, or even blackish appearance, are frequently met with; sometimes one portion of such a patch has already assumed a blackish color, while the rest still exhibits its greenish tint. In company with these patches, small plates of a triangular, quadrilateral, or rhomboidal form, or even minute rods, as well as large elongated pigment-granules, are often seen; they all show their crystalline nature by their heavy, dark contours, and their high degree of refraction (figs. 2 and 7). These elements, both fat-globules and crystalline plates, are not alone met with in tuberculous lungs, but also among the masses of black pigment in the alveolar septa of healthy ones. In the sections which I made from the healthy lung of a syphilitic woman, for instance, I thus observed not only a number of these elements, but also some minute crystalline rods. In examining closely the fat contained in the cells of any tissue affected by fatty infiltration or degeneration, it will be found that it does not always appear in the form of globules, but also in that of small oblong or even rod-like patches; this is especially the case in such portions of the organ, or neoplasm, where the circulation, and in consequence, the nutrition, have been interrupted for some time by the obliteration of the minute blood-vessels. Under such conditions an inspissation of the fat deposited in the cells takes place, which finally leads to the crystallization. This condition particularly occurs in tuberculous and leprous tissues. In miliary tubercle, as we know, the minute blood-vessels of the alveoli are obliterated at an early date of the pathological process by the proliferation of the tubercle-cells, which, for the want of nutrition, soon undergo fatty degeneration; they, moreover, by the continuous growth of the tubercle, become farther and farther removed from the supply of nutritive matters, as well as from the influence of the healthy tissues. It is under these circumstances that an inspissation of the fat which these cells contain, takes place, leading, finally, to the formation of the minute crystals; and, as the center of the tubercle is most remote from the healthy tissues, we would naturally expect the first crystals to be found in this place. But this appears not to be the case, for though not infrequently the crystalline rods, either single or in small groups, are met with in the center, and throughout the cheesy part of the tubercle, it nevertheless happens that they are found in much greater numbers along its margin. Though a reasonable explanation for this apparently contradictory phenomenon may be given, I must forbear at present from doing so, as it would lead me too far away from the main subject, and increase the length of this article. I therefore postpone the explanation of this phenomenon to some other occasion, and confine my remarks to stating, that I have also found comparatively large crystalline masses, or large groups of single rods and leaflets, in the indurated portions of tuberculous lung; they were unaccompanied by fat-globules, but always found in the vicinity of collections of pigment granules (fig. 6). These masses, when examined with oblique illumination, always resolve into an aggregation of minute rods, or needles. The same form of fatty crystals may be artificially produced, by means of the potash solution, in the sputa of tuberculous patients, or in any fattily infiltrated or degenerated tissue.

But the finest display of the minute crystalline rods, leaflets and needles I observed in the sections of a group of bronchial lymphatic glands, from a case of peri-bronchitis fibrosa. These glands, as is sufficiently known, contain a great deal of black pigment. The sections in question I had made and stained during last winter, and they had since been kept in a mixture of alcohol and water, but nevertheless showed their structure as neatly as if they had been cut the day before the examination. The microscopical examination of these sections, made after they had been treated with the potassa solution, revealed all the forms of the minute crystals above described; they were found throughout the tissue of the gland, but particularly in the vicinity of the abundant collections of black pigment, and between the groups of granules of the latter, all the transitory fatty and crystalline forms to which I have referred above, could here be thoroughly studied. After the section had been exposed to the action of the potassa solution for several hours, it was washed by a small current of water made to pass through it under the cover-glass; by this process the crystals were fully exposed to view, and appeared as they are represented in fig. 7. The most interesting part of the observations made on these sections, however, relates to the fat-cells met with in the areolar connective tissue which binds together the individual lymphatic glands of the group; a considerable number of which—at least one dozen in each section—were found to be completely filled with the minute rod or sword-like crystals of margaric acid. While in some of these cells all the crystals radiated from one common center, in others they radiated from the center of several large fat-globules contained within (fig. 3). In a number of instances the membranes of the cells had been opened by the knife, allowing a portion of the crystals contained within to escape and to present themselves to full view. Some of these sections were after the examination, subjected to the action of boiling ether, in the manner above described in connection with the sections of tuberculous lung, and when they were re-examined, it was found that *every crystal had disappeared*, and nothing but the empty cell-membranes were left (fig. 4). As in the sections of tuberculous tissue, the ether had dissolved the crystals.

Although the fattily degenerated cells are always met with in the tuberculous portion of a lung, they do not always contain pseudo-bacilli; it may happen, even, that while great numbers of these crystals are found in the sections made from a certain piece of tuberculous lung, none will be met with in other sections made from a neighboring piece of the same lung. In the sections made from different parts of the lungs of a case of disseminated miliary tubercle of eight months' standing, a boy fifteen years of age, I met with very few crystalline rods, though very numerous cells filled with fat-globules were present. The crystals here, together with the transitory forms which I have mentioned above, were chiefly found in company with groups of pigment-granules; this case, therefore, offered a favorable opportunity for the study of these transitory forms. There were numerous cavities, mostly small in size, and filled with a thin, glairy fluid found in the lungs of this case. In examining the fluid, a few hours after death, it was found that its viscidity was chiefly due to the presence of myriads of sphero-bacteria, and also small pus-corpuscles. The bacteria were clustered into innumerable minute balls, and many of them still in motion. In some of the sections, made from the border of a cavity, I observed quite a number of capillary vessels filled with numerous bacteria termo, which undoubtedly came from the cavity. But, as these organisms were obviously derived from the inspired air, there can be no significance attached to their presence.

The degenerated cells containing the pseudo-bacilli are, as before mentioned, not infrequently met with in the sputa of phthisical patients. I have, as yet, not had sufficient time to positively determine the exact route by which they get into the expectoration; for, though I have, in a few instances, met with some of them in apparently healthy alveoli, I do not think that they originated in these places. But, as they are frequently found in the close vicinity of minute cavities, and but rarely near the borders of a large cavern, we may presume that they are set free in the beginning of the breaking down of the tuberculous mass, when they may escape through a small bronchiole, laid open by the ulceration of the tubercle. The same characteristic cells, containing a small mass of pigment, with or without fat-globules or pseudo-bacilli, are also frequently met with in the expectoration. At any rate, the presence of these cells, either with or without the crystalline rods, or of single free rod-like crystals, always indicates the presence of minute tuberculous cavities in the lungs.

With regard to the mode of preparing the specimens of sputa, I may add, that they are simply spread as thinly and evenly as possible upon a glass slide, and left to dry; then one or two drops of the potash solution are put upon them, and the cover-glass applied. The particular strength of the solution is of little importance, for the stronger the solution the quicker the action, and *vice versa*. The same with the sections, which may before being mounted, be first exposed to the action of the solution, contained in a small porcelain capsule, or in a deep watch-glass. The time of exposure is about fifteen minutes for a 30 per cent., one hour for a 10 per cent., and twenty-four hours for a 1 per cent. solution. From the solution they are transferred to pure water, and from this, by means of a small spatula, to the slide. There the water is removed from the section by means of a moist camel's-hair brush, after which two or three drops of the potash solution are put upon it, and the cover-glass applied.

While I was pursuing the investigations described in the preceding pages, I often asked myself, whether the pseudo-bacilli which I observed were really identical with Koch's bacilli tuberculosis, or whether there existed some *true* bacilli, besides, in the tuberculous portions of lungs, which I had failed to discover. For I could hardly conceive the idea that one holding so prominent a position in the medical world as Dr. Koch, could commit the error of mistaking minute crystals of fatty acids for organized beings. But, as he has not mentioned in his treatise these crystals, which in so many points correspond to the description he has given of his bacillus tuberculosis, I must presume either that they have escaped his notice, or *that they are identical with the bacilli he described.*

I have already remarked, that the natural bluish color, which these minute crystals exhibit, may be taken for true staining with methyl-violet; and I have also stated that if sections of tuberculous lung, containing the pseudo-bacilli, are, after treatment with the solution of caustic potassa, stained *only* with vesuvin, and then mounted in Canada balsam, *these bodies will show the same blue tint as if the sections had previously been stained with methyl-violet.* My suspicions that Koch's bacilli had never been stained, were considerably strengthened when I read the remarks made some time ago by the Berlin correspondent of the Philadelphia *Medical News*, who, though apparently a tyro in scientific microscopical matters, is an enthusiastic admirer of the bacillus tuberculosis. This gentleman, in describing the method of preparing the specimen for the demonstration of the bacilli tuberculosis, says: * "The specimen may be conserved in the usual way with Canada balsam, but it is important to know that *the color fades out in about two months.* It may be restored, of course, by beginning *de novo*, after careful recovery of the specimen from its bed of balsam with turpentine in the usual way.

"Some one asked Dr. Koch, the other day, why he did not prepare and sell the specimens, and thus realize a small fortune. He would consider such a work unworthy the dignity of a science, he said, but even if he had not been restrained by any such feelings, he would have rendered himself liable to the charge of imposture, he added, with a smile, after the color of the specimens had faded out."

Here we have, then, Koch's own statement that his bacilli tuberculosis part with the color which they are said to absorb by the staining process. Now, true bacilli never part with the methyl-violet, having once absorbed it, but, like micrococci, or other vegetable tissues, retain it for years. This I know from experience. And often have I convinced myself of the greediness with which bacilli absorb methyl-violet, by simply stepping from my office to the street gutter, and procuring a portion of the rainbow-colored film which rests, almost throughout the whole year, upon the surface of the standing gutter-water. This film, when examined under the microscope, is found to be crowded with minute organisms, such as infusoria, algæ, and the whole list of schizomycetes, from the sphero-bacteria up to the spirochæta. When a small portion of this film is mixed with a weak solution of

* The *Medical News*, August 12, 1882, p. 193.

methyl-violet, and about ten minutes afterward examined under the microscope, it will be found that all the bacteria, among which is the bacillus subtilis, as well as the infusoria, are now colored purplish-blue, and that in proportion to the length of time during which they are exposed to the color the latter increases in intensity. A refusal of true bacilli to absorb fine coloring matters, such as methyl-violet, could only be explained by supposing them to possess, like diatomacæ, a silicious coating. To assert, however, that the bacillus lepræ and bacillus tuberculosis do so differ from other bacilli as to possess such a coating, would require a very considerable stretch of the imagination.

Another instance of the early disappearance of the bacillus tuberculosis from the preparations, was communicated to me a few days ago in the following way: Some weeks since I learned, through one of our medical journals, that a certain prominent American physician had just returned from Germany, and brought with him several preparations of the bacillus tuberculosis from Koch's laboratory, which, at his arrival, he had exhibited to some medical society. Being very anxious to examine some of these specimens, for the purpose of comparing Koch's genuine bacillus tuberculosis with my crystalline pseudo-bacillus, I addressed a letter to this gentleman, in which I requested him to afford me this opportunity by lending me one of his specimens for a few days. My request was kindly granted. But, while I was anxiously looking for the arrival of the specimen, the doctor informed me by a second letter, "that all his specimens from Koch's laboratory had lost color, and hence the bacilli were invisible."

They had become invisible, because they had never been stained, though their natural bluish tint, with their contours, may have been distinguishable when the specimen was first mounted.

But let us try to find an explanation for this early disappearance of these crystalline rods from the balsam mounting. I have already alluded to the intermediate forms observed between the simple fat-globules and the crystals, or even the pigment, which is also crystalline in nature. Now, in examining, especially, the rod-like crystals a little closer, it will be found, that though they are all refractive in a high degree, their contours differ in the different rods in sharpness, heaviness and darkness ; for, while some of the rods are darkly and heavily contoured, the borders of others are marked by finer lines ; the latter, also, present a brighter, more greenish tint. Now, it may be presumed, that this difference existing in the contour, indicates a difference in the degree of crystallization of the rods; the darker and more pronounced the contours the higher is the degree of crystallization, and at the same time, the greater the power of resisting solving agents. Accordingly I observed, during the re-examination of those sections of tuberculous lung which were boiled in ether, as described before, that, though the rods, in general, had disappeared, a few single ones—perhaps one or two in a hundred—exhibiting very dark and heavy contours, were here and there met with, having resisted the solving power of the ether. They might, perhaps, have finally dissolved, if the boiling in ether had been continued for a longer time. The early disappearance of Koch's bacilli in the balsam mounting may take place under similar circumstances, namely, they may be acted upon by the solving power of the benzol, which keeps the balsam in solution ; though the increasing transparency of the balsam may also play a part in the phenomenon.

After the perusal of the facts brought forward in the preceding pages, the reader may, perhaps, ask : If this bacillus tuberculosis represents the minute crystal of a fatty acid, whence, then, came the bacilli which Koch obtained by cultivation from the sputa of phthisical patients, as well as from the tuberculous matter of the lungs? This question I can only answer by replying, that I attach no signification whatever to the presence of bacilli, or other minute organisms in the expectoration of tuberculous patients, because the germs of these organisms have, with each inspiration, ready access to the cavities of the lungs. It is a common occurrence to meet with micrococci, even, in the sputa of non-tuberculous patients, but more frequently still in that of phthisical patients. In some specimens of tuberculous sputa, I have even observed numerous bacilli subtilis, which, as I have stated before, are constantly found in our street gutters. Therefore, the assertion of any minute organism being the cause of tuberculosis is invalid, as long as this organism is not met with in the blood, or in the tuberculous portions of the lung, and in such numbers as will render its noxious influence evident. As regards the bacilli which Koch raised from tuberculous matter, I can only say, that I always regarded with suspicion the organisms obtained by culture from pathological tissues or secretions, for the obvious reason, that their germs appear to be omnipresent, and the experimenter hardly knows himself the origin of all the bacteria which he finds in his culture-cell, or upon his culture-substance. The same remarks may be applied to the results of the inoculation experiments made on various animals, for the statements of the results of quite a number of experimenters, recorded in the literature on this subject, are so contradictory as to render it difficult to decide what is true and genuine.

Although there remains much more to be said on the subject of tuberculosis being caused by minute organisms, I must postpone all further remarks to some other, more opportune time, when I shall be more at leisure to continue the discussion on this interesting subject.

In connection with the bacillus tuberculosis I have a few remarks to make on its near kin, the *bacillus lepræ.* To find this bacillus I set about, nearly a year ago, and stained very thoroughly a number of sections of various leprous organs with methyl-violet. In examining these, mounted in Canada balsam, however, I failed to discover anything resembling a bacillus. Thinking it strange that I should have failed to see this bacillus, which from the statements of other observers, ought to have been present in these sections, I wrote a paper entitled: "Is the Bacillus Lepræ a Reality or a Fiction?" in which, beside reviewing the statements of other observers, I stated the results of my own examinations, and the method of staining I had employed. The paper was published in the CHICAGO MEDICAL JOURNAL AND EXAMINER, April, 1882. Two months afterward, a paper entitled "The Bacillus Lepræ," by Dr. I. Bermann, of Baltimore, appeared in the *Archives of Medicine*, in which the author winds up the subject as follows: "In the April number of the CHICAGO MEDICAL JOURNAL AND EXAMINER, Dr. H. D. Schmidt, of New Orleans, discusses the question whether the bacillus lepræ is a reality or a fiction. That he has not succeeded in finding them in his specimens I cannot doubt, since it was testified that they could not be seen in them by microscopists in Chicago. I am satisfied that some fault in his method is alone the cause of his non-success, and I should be very glad to stain some of his material if he will send to me, and believe that I could convince him in this way of the unfictional character of the bacillus lepræ."

Now, notwithstanding that I have made double and triple stainings of thousands of sections, both of animal and vegetable tissue, and accordingly gained some experience in the various methods of staining, I am still ready to learn from others, even from my own pupils; and therefore, soon after reading the above remarks of Dr. Bermann, I complied with his request, and sent him a number of my leprous sections made from different organs of different cases, so that he might try his skill in staining the bacilli, which these tissues are said to contain. Some weeks after this, he informed me by letter that he had succeeded in staining the bacilli beautifully, but that in all of them the color had disappeared after three or four days, a circumstance which he attributed to the presence of the picric acid in the picro-carmine with which the sections had been stained, and that for this reason, he would try again to stain the bacilli by previously treating the sections with a strong alkali. At the same time, in order to convince me of the existence of the bacillus lepræ, he offered to send me a preparation of his own, still living, case. This was the last which I heard from Dr. Bermann. Even a letter addressed to him five weeks ago, in which I expressed the desire of beholding the bacillus lepræ, and furthermore, reminded him of his promise to send me one of his own preparations, remained unanswered. I must, therefore, presume that the bacilli lepræ in his own preparations have disappeared from view in the same manner as the bacilli tuberculosis of Koch, for the reason that they never had been stained. And that this supposition is based upon facts observed, I shall now show.

After I had discovered the minute crystals, described above, in the tuberculous lungs, I proceeded to treat a number of sections of leprous tissues from different organs and cases with the solution of caustic potassa, and, as I anticipated, met here with the same highly refractive rod, lancet, and needle-shaped crystalline bodies, exhibiting the same blue tint as the former. As in the lungs, they were always found associated with fat-globules, and in many instances, even, seen to arise from them in the same manner as is observed in the crystals artificially produced by the action of caustic potassa upon tuberculous sputa (fig. 9, *b*). As in the case of the pseudo-bacilli tuberculosis, the same transitory forms, intermediate between the fat-globules and the crystals, and indicating the different degrees of crystallization, were here met with. Among these forms, a number of minute knotted or varicose rods, which evidently showed that they had been formed from a few very minute fat-globules fused into each other (fig. 9, *a*), were also observed. In the sections of leprous livers, also, many opaque-looking cells, containing a considerable number of these crystalline bodies in company with fat-globules were observed, though in many other places in the sections, single rod or lancet-shaped crystals, or groups, formed by two or three of them, associated with fat-globules, were likewise met with.

In order to make sure of the crystalline nature of these bodies, I treated a number of sections of liver, lymphatic glands, skin, etc., from different cases, with the potash solution ; and after having convinced myself by microscopical examination that they contained the crystalline bodies, they were boiled in ether, in the manner described in connection with the sections of tuberculous lung. By re-examining these sections under the microscope, it was found that *every crystal had disappeared from view.*

In my paper on the bacillus lepræ, mentioned above, I stated that in my former studies of leprous tissues, I had met with a number of degenerated neoplastic cells (originally derived from the nuclei of the fat-cells lodged in the reticulated portion of the corium and the subcutaneous layer of the skin) filled with minute crystals of margaric acid; and furthermore, remarked that in sections not stained with hematoxylin or methyl-violet, these crystals, though easily recognized, might nevertheless be taken for bacilli. Rather thinking, at that time, that those small, very slender nuclei met with in the corium of the skin, were taken for bacilli, I little imagined that the remarks I made in regard to the crystals would come so near the truth. And now, after having more thoroughly investigated this subject, I can hardly understand how these minute rod, lancet, and needle-shaped crystals could ever have been taken for true bacilli, characterized by their blunt, almost square extremities.

### EXPLANATION OF THE ILLUSTRATIONS.

Fig. 1 represents a small portion of the margin of a tubercle. The section has been treated with the solution of potassa, which has rendered the tubercle-cells pale and indistinct. A number of fattily degenerated, opaque cells, containing the fatty acid crystals with fat-globules, are seen in two of the alveoli; their protoplasm has also been rendered clear by the action of the potassa solution, so that the various forms of the crystals are distinctly seen. Near the elastic fibers of the alveolar septa, the remains of degenerated cells, in the form of groups of crystals associated with black pigment, are seen. On the left side of the figure, the cheesy portion of the tubercle commences.

Fig. 2 represents an alveolar from a section of tuberculous lung, which had been exposed to the action of the potassa solution, until the protoplasm of the cells was completely destroyed, and the crystals exposed to full view.

Fig. 3 represents three fat-cells from the connective tissue, binding together a group of bronchial lymphatic glands; they are filled with fatty-acid crystals, as mentioned in the text.

Fig. 4 represents the empty membranes of some of these cells, after the boiling of the section in ether.

Fig. 5 represents two fat-cells from the vicinity of the preceding cells, in which the fat appears to have been converted into pigment.

Fig. 6 represents various forms of groups of satty-acid crystals, met with in the section taken from an indurated portion of the tuberculous lung bordering a cavern.

Fig. 7 represents a small portion of a section of a bronchial lymphatic gland, mentioned in the text. The glandular parenchyma has been destroyed by the action of the potash solution, exposing the crystals to full view.

Fig. 8 represents the fatty acid crystals arising from patches and globules of fat in tuberculous sputa, treated with a solution of caustic potash.

Fig. 9. Pseudo-bacilli lepræ; *a*, different forms of the crystals; *b*, two hepatic cells, in which the pseudo-bacilli are seen to arise from fat-globules; *c*, remains of a hepatic cell, with the pseudo-bacilli lying between the fat-globules; *d*, hepatic cell containing very minute lancet-shaped crystals with minute fat-globules; when illuminated with oblique light, they all appear very highly refractive.

All the figures are magnified about 525 diameters.

NEW ORLEANS, Nov. 10, 1882.

## Figures and Tables

**Fig. 1 Fig. 2 Fig. 3 Fig. 4 Fig. 5 Fig. 6 Fig. 7 Fig. 8 Fig. 9. f1:**